# Climate change effects on the Baltic Sea borderland between land and sea

**DOI:** 10.1007/s13280-014-0586-8

**Published:** 2015-01-09

**Authors:** Alma Strandmark, Arvid Bring, Sara A. O. Cousins, Georgia Destouni, Hans Kautsky, Gundula Kolb, Maricela de la Torre-Castro, Peter A. Hambäck

**Affiliations:** 1 Department of Ecology, Environment and Plant Sciences, Stockholm University, 106 91 Stockholm, Sweden; 2 Water Systems Analysis Group, Institute for the Study of Earth, Oceans and Space, University of New Hampshire, 8 College Road, Durham, NH 03824 USA; 3 Department of Physical Geography and Quaternary Geology, Stockholm University, 106 91 Stockholm, Sweden

**Keywords:** Baltic Sea, Climate change, Coastal ecosystems, Sea shores, Sea level, Coastal management

## Abstract

Coastal habitats are situated on the border between land and sea, and ecosystem structure and functioning is influenced by both marine and terrestrial processes. Despite this, most scientific studies and monitoring are conducted either with a terrestrial or an aquatic focus. To address issues concerning climate change impacts in coastal areas, a cross-ecosystem approach is necessary. Since habitats along the Baltic coastlines vary in hydrology, natural geography, and ecology, climate change projections for Baltic shore ecosystems are bound to be highly speculative. Societal responses to climate change in the Baltic coastal ecosystems should have an ecosystem approach and match the biophysical realities of the Baltic Sea area. Knowledge about ecosystem processes and their responses to a changing climate should be integrated within the decision process, both locally and nationally, in order to increase the awareness of, and to prepare for climate change impacts in coastal areas of the Baltic Sea.

## Introduction

Climate change may affect seashores in several more ways than inland habitats, including effects of rising sea levels, changed wind patterns, and reduced ice cover. Sea level rise has, since the 1960s, been caused by a combination of thermal expansion of the sea and melting ice packs, each accounting for about half of the increase (Church and White 2011). Sea levels are expected to increase at an even higher rate in the future (Church et al. 2013). Projected changes in wind patterns will alter conditions for seashore organisms, and a reduced ice cover in northern areas will influence the occurrence, timing, and intensity of ice scouring. Ice scouring is an important process shaping many coastal habitats in the northern and central Baltic Sea.

For terrestrial plants and animals, conditions on seashores are often stressful, due to saline soils, wave action, and currents, and many species show special adaptations. Plants on the sea shore, for example, can have increased abilities to excrete salt in leafs or roots compared to plants in other terrestrial habitats, and seeds may preferentially germinate in periods with a high inflow of freshwater (Jerling 1999). Marine plants and animals, on the other hand, must survive conditions at low tide when they are also exposed to high predation rates from terrestrial predators. Seashores are typically species-rich in both plants and arthropods (Ievinsh 2006) and may be visited by large numbers of birds during migration and over-wintering. At the same time, many people live on or close to the sea and use seashores for many purposes. Consequently, coastal areas are key targets for conservation and considerable efforts are spent in preserving coastal reserves from beach erosion and human encroachments. The so-called coastal squeeze, between rising sea levels and human settlements, will in many places limit the ability of plants and animals to move upland in response to increasing sea levels, thus the effect of climate change on sea shore habitats may be more profound compared to other habitats. While climate scenarios are developed to describe global projected changes, effects of climate change are likely to vary around the world (Church et al. 2004), even within the same region (Bring and Destouni 2013). In the Baltic Sea, climate change may have unique consequences for several processes and environments, and the ecological responses are likely to vary within the region.

**Fig. 1:**
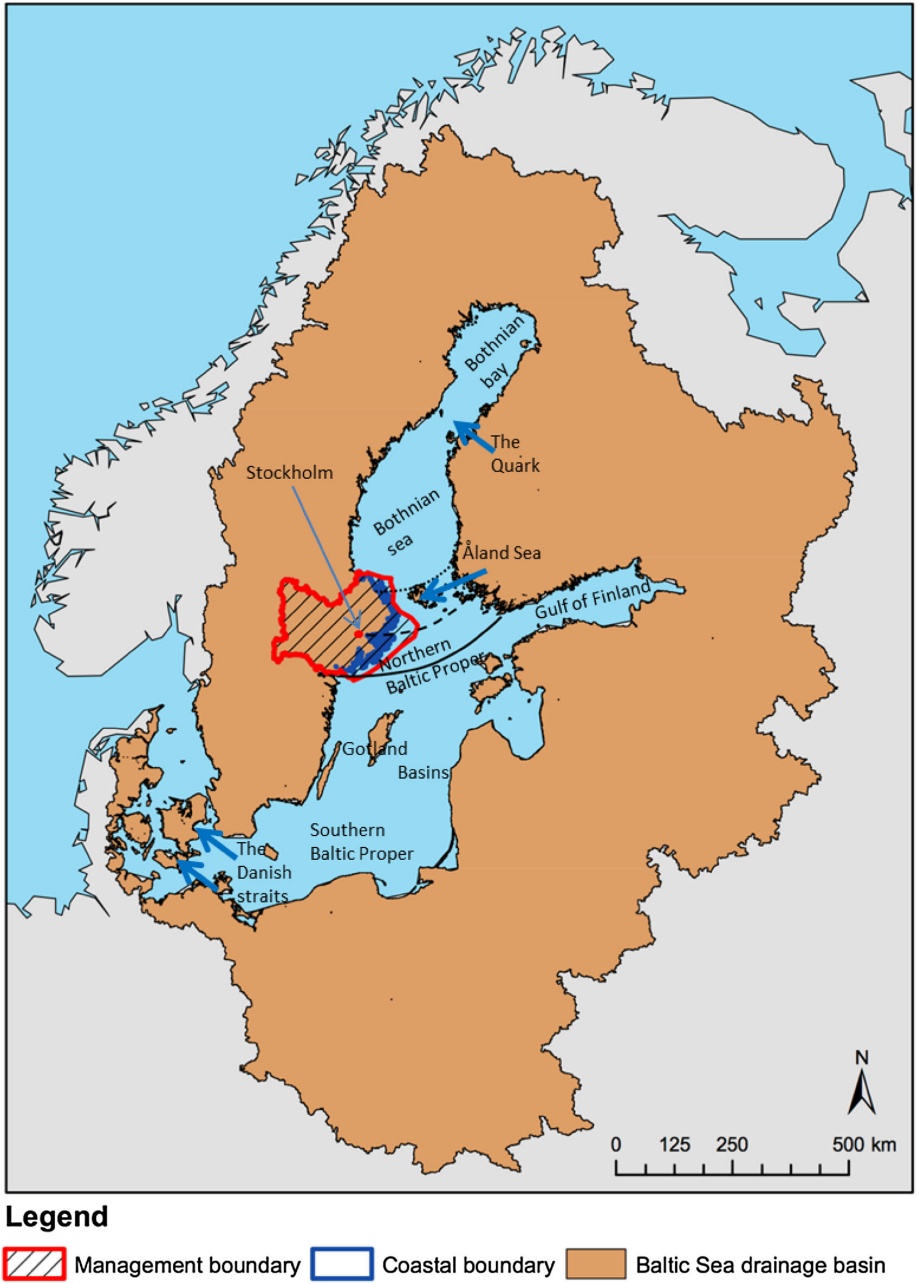
Baltic Sea drainage basin (shown in *brown*) with subareas, here including the Danish Straits and Kattegatt. An example of a water management district in Sweden, the Northern Baltic Proper, is *colored* *red*, with the coastal boundary in *blue*. The example of water management district, administratively formed as part of the national Swedish implementation of the EU Water Framework Directive, illustrates that the coastal water falls within the terrestrial domain of water management, with the district boundary extending between 5 and 50 km from the coast. *Lines* across the Baltic Sea illustrate the approximate position of the line of equilibrium between isostatic rebound and SLR, for the present situation of a sea level rise rate of around 3 mm year^−1^ (*solid* *line*), and potential future higher rates of 4 mm year^−1^ (*dashed* *line*) and 5 mm year^−1^ (*dotted* *line*)

Since the last Ice Age, 10 000 years ago, isostatic rebound has caused the shores of the Baltic Sea to fall in conjunction with land uplift (Ekman 1996). The highest rate of isostatic rebound, in the Bothnian Sea (Fig. 1), is close to 1 cm per year, presently outpacing most of the projected range of sea level rise (SLR) for the Baltic Sea. At the most extreme climate scenarios, however, the uplift rate will not compensate for the SLR by the end of the century. The constant renewal of the shoreline caused by the land uplift is a natural process that has occurred for thousands of years. Since both vegetation and landscapes on most Baltic Sea shores are strongly shaped by land uplift and the continuous colonization of new land, the conditions will fundamentally change if this process is reversed. The Baltic Sea is unique for its sharp latitudinal salinity gradient. Large inflows of freshwater from the rivers in the north reduce the salinity, while saltwater inflows through the Danish straits from the North Sea increase the salinity in the south. Ice scouring is another important shore process in the northern and central parts of the Baltic that most likely will change in occurrence and intensity due to a combination of SLR and warmer winters. These features make the Baltic Sea a particularly interesting case for exploring climate change effects on shores.

In this paper, we combine information from physical geography, hydrology and terrestrial and marine ecology to discuss climate change projections and the impacts on shore ecosystems along the Swedish Baltic Sea coast. As coastal ecosystems in this area are intensively used by people, and human-related development may limit ecological adaptations to climate change, we include a societal perspective on climate adaptations. In our view, a cross-disciplinary approach is necessary to understand the present and future dynamics in the borderland between land and sea. We focus on the central Baltic Sea region where the postglacial land uplift is predicted to be reversed in the near future even with moderate SLR (the area between the current equilibrium line and the projected future line Fig. 1). Although the focus of the paper is on terrestrial shore ecosystems, climate change impact on marine near-shore habitats will also be discussed since the boundary between the two elements is far from discrete. To accurately project climate change effects on shore ecosystems, it is important to understand how processes in different parts of the littoral zone affect each other.

First, we describe the current conditions and projected changes in sea level, salinity, and ice cover in the Baltic Sea. Second, we describe the Swedish society’s response to climate change, and third, the ecological processes determining the plant and animal community on Baltic seashores. Then, we describe the processes affecting the marine system and how they are linked to terrestrial shore ecosystems. Finally, we combine these processes and responses to project future consequences of climate change in Baltic shore ecosystems and discuss which societal actions are needed for better awareness and adaptation at the land–sea interface.

## Dynamic changes at the coastal boundary

The coastal boundary is subject to continuous geophysical and biogeochemical changes. These changes and their drivers act on various time scales. For instance, sea level change in the Baltic, a major control on coastal geography, ecology, and water dynamics, is principally determined by three factors: the postglacial isostatic rebound of land, the global eustatic SLR due to present global warming, and the water balance of the Baltic Sea (Johansson et al. 2003). The first two processes are multi-centennial in nature. The variability of the last process, on annual and longer timescales, correlates significantly with the large-scale climate pattern termed the North Atlantic Oscillation (Kahma 1999; Johansson et al. 2003). In the southern Baltic, the long-term mean land uplift rate is now counterbalanced by the global SLR (Fig. 1), where the latter rate is presently about 3 mm year^−1^ (Church and White 2011). Assuming the global average SLR as representative also for the Baltic, Meier et al. (2004a) simulated future Baltic sea level change. Based on scenario estimates in the Third Assessment Report of the IPCC (Church et al. 2001), Meier et al. (2004a) arrived at winter net sea level changes at Stockholm of between −480 and 460 mm based on a global SLR of between 90 and 880 mm until year 2100. The IPCC assessed that the rate is very likely to increase during the twenty first century (Church et al. 2013) and in the Fifth Assessment Report, the estimates of global SLR until 2100 vary from 260 to 970 mm for different emission scenarios (Church et al. 2013). Some recent estimates have arrived at even higher SLR values; for instance, the Arctic Monitoring and Assessment Program (AMAP 2012) estimated a likely upper limit of 1600 mm by 2100. It is thus reasonable to consider the upper range of recent SLR projections, which implies a northward shift in the line of equilibrium in the Baltic Sea between the isostatic rebound and SLR. Currently, this equilibrium line crosses the Baltic Sea in a west–east-directed arch from Norrköping in Sweden to Hanko in Finland (Fig. 1). Today shore erosion is a problem in areas south of this line (Sterr 2008), which will be enhanced with further increased sea levels.

**Fig. 2:**
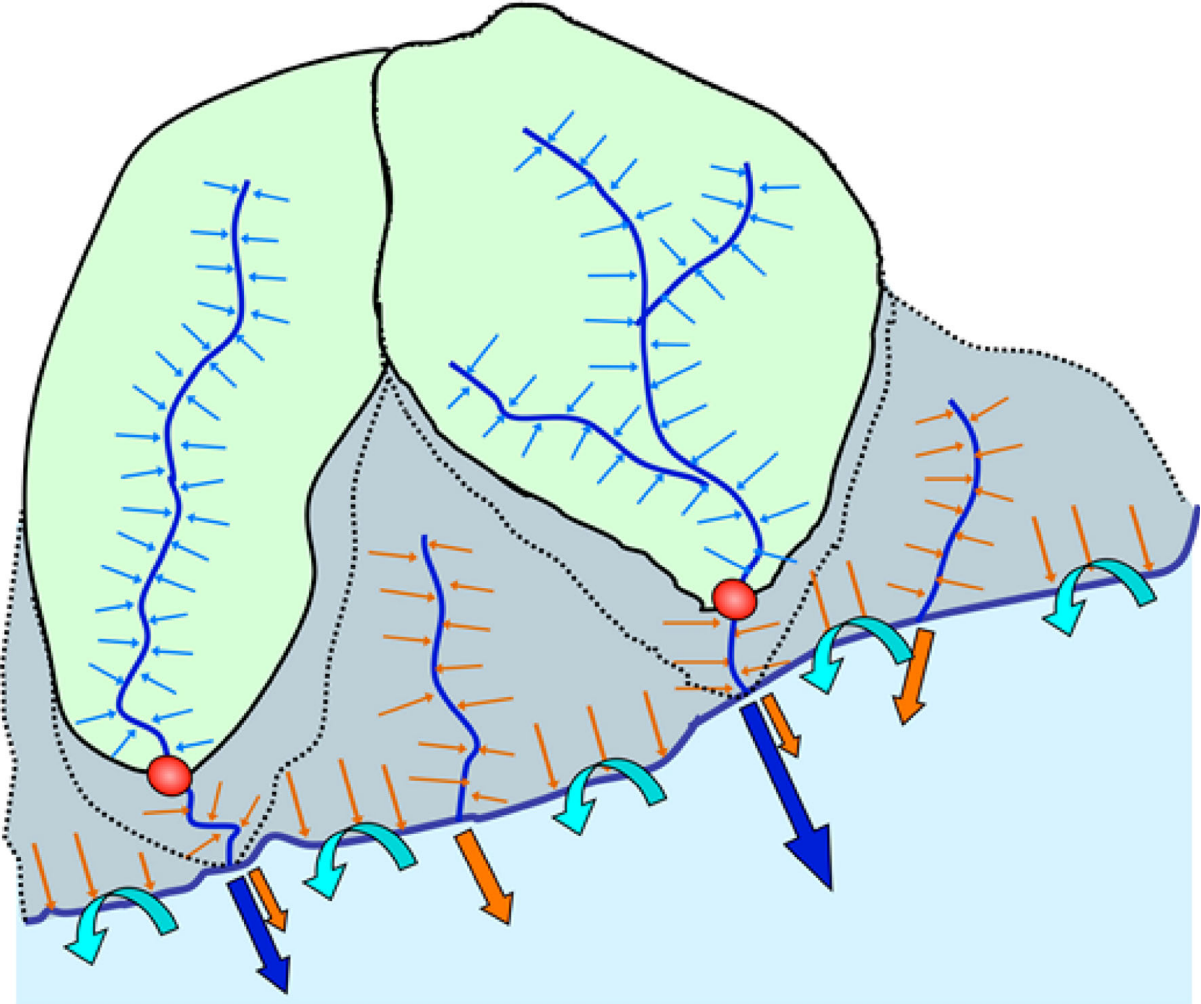
Schematic illustration of monitored and unmonitored pathways of water flow and waterborne nutrient/pollutant transport across the coastal boundary. *Solid* and *dotted* *black* *lines* are water divides of monitored (*green*) and unmonitored (*gray*) parts of coastal catchments. *Red*-*filled* *circles* show the most-near coastal monitoring stations that define these catchment parts. *Straight* *flow* *arrows* at and across the coastline boundary illustrate monitored (*blue* *flow* *arrows*) and unmonitored (*orange* *flow* *arrows*) freshwater discharges from land to sea. *Turquoise* *curved* *flow* arrows across the coastline boundary illustrate the re-circulated seawater component of submarine groundwater discharge. *Blue* *lines* within the catchments show rivers and streams, and *blue* and *orange* *flow* *arrows* into them illustrate the groundwater flow into monitored rivers and unmonitored streams, respectively. Modified from Destouni et al. (2008)

Net changes in sea level also interact with other geophysical and biogeochemical processes along the coast. For instance, increased seawater intrusion into coastal groundwater may result from rising sea levels, as well as decreasing flow of fresh groundwater to the coast (Mazi et al. 2013). The water discharges across the coastal boundary are complex combinations and mixtures of water flow through various pathways (Fig. 2), implying a risk of underestimating the total land-to-sea fluxes (Destouni et al. 2008). Climate change will have consequences for the different water discharges (Destouni et al. 2013), soil water dynamics (Destouni and Verrot 2014), and waterborne nutrient and pollutant loads from land to the sea (Darracq et al. 2005), which will in turn also present society with significant management challenges in the coastal zone.

Other important biogeophysical determinants for the Baltic Sea coastal boundary include winter ice cover, storms, and salinity. In contrast to the widely acknowledged IPCC scenarios, no distinctively accepted scenarios exist for future changes of these determinants.

Changes in salinity have recently been summarized by Heino et al. (2008) and HELCOM (2013). Present salinity averages about 7.7 psu (practical salinity unit, equivalent to per mille or to g/kg) over the entire volume of the Baltic (Heino et al. 2008) and is primarily governed by runoff from land and wind patterns. Projections indicate a decreased salinity due to increased precipitation, and corresponding runoff. However, the projections are uncertain since global runoff forecasts by the IPCC must be interpreted with caution due to a high runoff variability over the Baltic Sea region (Fig. 12.24 in Collins et al. 2013). Runoff may also be influenced by changing land use (Destouni et al. 2013) in conjunction with changed soil moisture variability (Verrot and Destouni 2015).

**Fig. 3:**
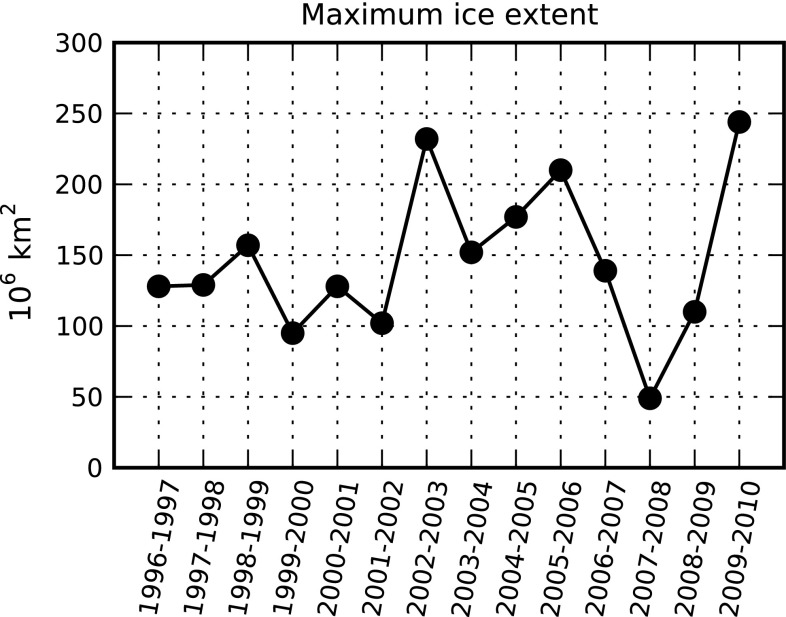
Inter-annual variability of maximum extent of sea ice-sheet in the Baltic Sea between 1996 and 2004. Data from Baltic Sea portal (http://www.itameriportaali.fi/en/tietoa/jaa/jaatalvi/en_GB/2010/)

In comparison to salinity changes, changes in ice cover are projected with greater certainty. A large reduction in length of the ice-cover season, particularly in the central Baltic, is projected by several models (Graham et al. 2008). Experiments with the regional climate model RCAO suggest that average ice extent may decrease by 57–71 % by the end of the century (Meier et al. 2004b). The inter-annual variability in ice cover is large in the Baltic Sea (Fig. 3), and the most noticeable change will likely be a decrease in the frequency of severe ice winters. Regarding wind speed changes, results from regional climate model simulations vary substantially with the global climate model used to drive the regional climate modeling (Graham et al. 2008). Some relatively robust changes include a greater increase in average winter wind speed over the northern Baltic Sea (Fig. 3.24 in Graham et al. 2008). In contrast, projected summer wind speeds show opposite signs for different models (Fig. 3.25 in Graham et al. 2008).

The changes in the biogeophysical drivers described above will alter ecosystem conditions at the land–sea interface within the present century, at a rate that is likely to increase, and thus increase the need for societal responses in coastal management. We first review these societal responses and then return to ecosystem effects in the subsequent sections.

## Societal responses in Sweden

There are a wide variety of societal responses to climate change and to sea shore changes in particular, but a clear and holistic consideration of land–sea interactions is still missing. The governance structure for environmental and land/shore protection in Sweden is complex involving several levels (national, regional, and local) with different actors playing different roles. At least 30 sectorial government agencies deal with climate-change-related issues. The national environmental objectives, decided by the parliament are implemented by several different agencies, including the Swedish Environmental Protection Agency (www.miljomal.se). One of the main goals is “Reduced Climate Impact,” introduced in 2009, with the aim not to exceed the increase 2°C temperature threshold.

In the Baltic, the HELCOM cooperation deals with the effects of climate change and also with shore protection. In Sweden, the Swedish Agency for Marine and Water Management is in charge of these issues, although specific problems have to be handled with the municipalities involved. For instance, flooding is of crucial concern within municipalities, especially in southern Sweden where the awareness and emergency preparedness have increased after recent flooding events (SKL 2009). Municipalities have been clear about their responsibility (SKL 2011) with a commitment to handle effects of climate change. But there is also a need for involvement at the national level, particularly to deal with the lack of economic and human resources as well as information base (SKL 2010). For this purpose, a portal about climate adaptation has been created within the Swedish Meteorological and Hydrological Institute (SMHI) as part of the National Knowledge Centre for Climate Change Adaptation (www.klimatanpassning.se). This initiative aims to provide agencies and citizens with concrete tools for adaptation to climate change. The manual for sustainable development of beaches (Rydell et al. 2011) presents an integrative view considering technical issues, environment conditions, expected changes as well as economic aspects. The manual specifically deals with potential catastrophic natural events such as the risks of severe erosion, land-slides, and flooding.

In general, societal responses are relatively recent and still under development. However, there is a lot of activity addressing climate change challenges at all levels of the Swedish governance system. Non-governmental organizations, such as the Nature Conservation Society, play active roles creating opinion, commenting on government initiatives, and involving the public. As erosion effects on beaches have become more apparent, massmedia has also become more active in reporting such problems. As the effects of climate change on beaches have been analyzed and highlighted by scientists, media is likely to continue reporting and indirectly creating increasing awareness and demand for responses. Media usually focus on climate change effects caused by increasing temperatures and not on the processes, species, and ecosystems that are affected.

Although there is an increasing awareness of climate change issues, societal responses are often conducted without synchronization of knowledge from different disciplines. Baltic shores and shore ecosystems are highly heterogeneous and thus societal responses will need to adapt to local conditions.

## Terrestrial shore ecosystems in the Baltic Sea

The development of the Baltic shoreline is the result of many glaciations and sea level changes and continues to change because of the isostatic rebound. Thus, the shore ecosystems in the Baltic Sea are characterized by rapid physical changes in both time and space.

**Fig. 4:**
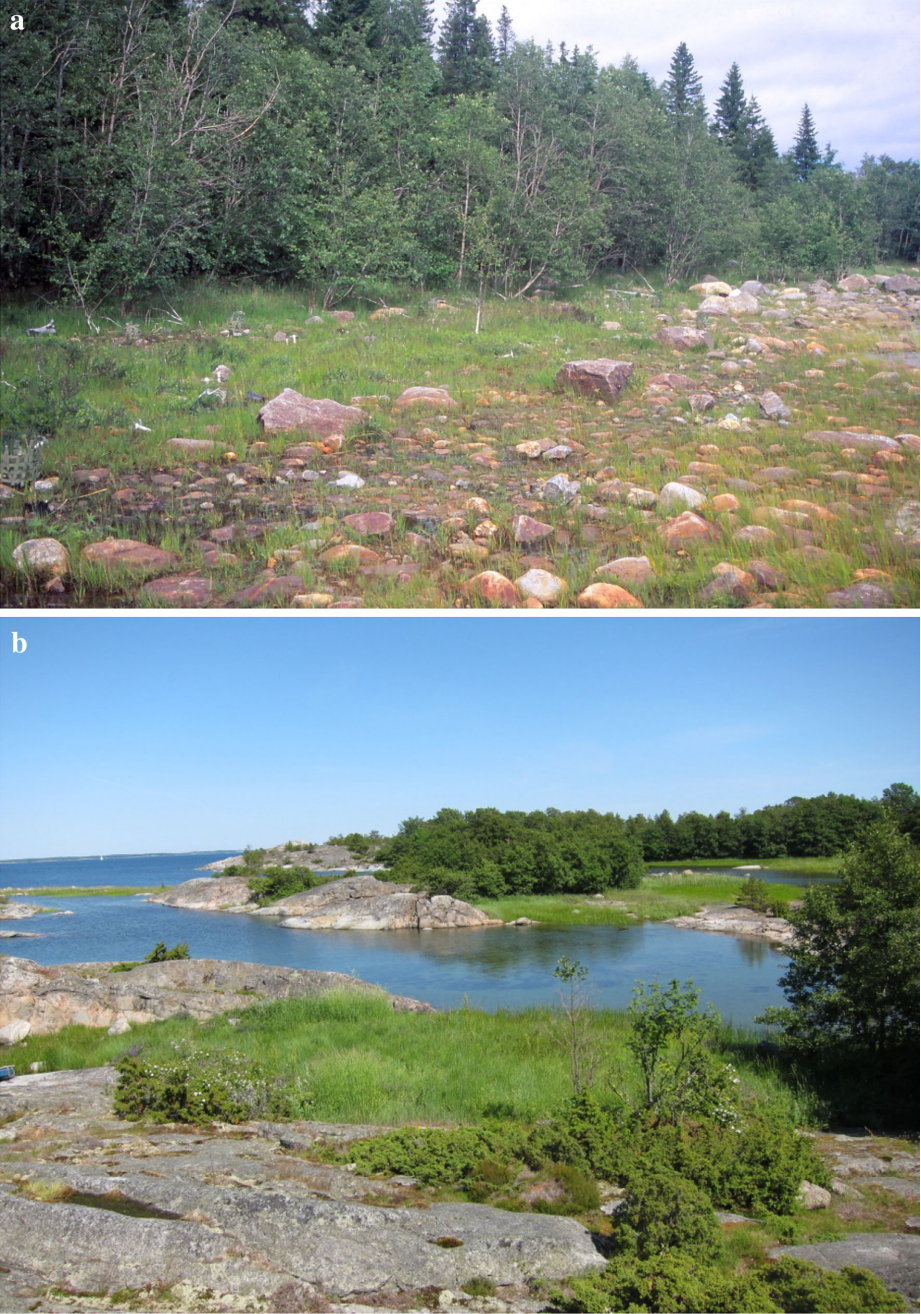
**a** A typical shore habitat in the Bothnian Bay. Vegetation is usually low and signs of ice scouring can be seen on the trees; **b** The typical fragmented landscape in a Baltic archipelago around Stockholm. Small shore meadows are bordered by forest, bedrock, or open water

Shores along the Baltic Sea vary greatly both among and within regions, but some general patterns are apparent. North of Åland, Swedish shores are typically flat and heavily ice scoured during winter (Fig. 4a), particularly in exposed areas. The long flat shores show a distinct successional gradient from grasses close to the water and herbs and shrubs closer to the forest (Ericson 1980). South of Åland, particularly in the area around Stockholm, rocky shores are common with little or no loose material. Plant communities typically vary with the steepness of the shore. On rocky, steep slopes, where wave action removes finer materials, lichens dominate, with taller plants in crevices (Jerling 1999). On more protected shores, where organic and fine-grained inorganic material has accumulated, the vegetation is more diverse, including species-rich shore meadows (Fig. 4b). Relatively flat shore meadows were used for grazing for a very long time, but today few shore meadows are managed. Since managed shore meadows have a much higher number of plant species than abandoned meadows (Cousins et al. 2015), ceased grazing has reduced species diversity.

Ecke and Rydin (2000) found that the early colonizers on uplift coasts are dominated by clonal species and stress-tolerators that quickly recover from physical disturbances, such as ice erosion (Jerling 1999). Grasses dominate close to the sea, whereas herbs are found further up where soils are drier and physical disturbance and salinity are lower. Higher up on the shore, there is an establishment of woody plants and particularly nitrogen-fixing species, such as alder (*Alnus glutinosa*), sea-buckthorn (*Hippophaë rhamnoides*), and sweet gale (*Myrica gale*). Nitrogen-fixing species are particularly important as they have a nutrient-rich litter that may facilitate establishment of other plants.

Because the plant community succession, and indirectly the arthropod community, on many Baltic shores depends on the establishment of bare soils caused by the isostatic rebound, it is expected that a reversal of this process due to SLR would have dramatic consequences for shore plants and animals. Moreover, increased sea levels may further diminish many already small and isolated species-rich shore meadows that today function as refuge habitats for plants, following the last century of grassland abandonment (Löfgren and Jerling 2002; Cousins et al. 2015). When shore meadows are diminished, connectivity between suitable habitats for many shore organisms will decrease together with ecosystem resilience (Auffret et al. 2015). Upwards migration of shoreline plants may be limited because land uplift and wave action have concentrated fertile habitats to low lying parts of the shores. The combination of eutrophication and cessation of grazing together with climate change is a great threat to the plant species diversity of Baltic Sea shores (Aggemyr and Cousins 2012).

Rising sea levels, with larger and more frequent flooding events, may also change the occurrence of the freshwater inflow in spring, which is necessary for many shore plants to germinate in coastal ecosystems (Jerling 1999). Although flooding keeps shore meadows open by restricting development of higher vegetation, flooding during the wrong time of year (during some critical point of development) and during too long periods (creating long periods of oxygen depletion in the soil) might be negative for the survival of seashore plants and their associated insects.

For terrestrial arthropods, Baltic shores represent a fragmented landscape with a complex mixture of suitable habitats interspersed within a matrix of rocks, forests, and water. Arthropods are mainly found in places with vegetation, primarily in shore meadows which host a large number of species, including many threatened ones (Ievinsh 2006). The arthropod community structure on shores is very different from more terrestrial communities. Several ecological factors may underlie this difference, both connected to plant community structure and to physical factors such as wind and ice scouring. The large inflow of marine nutrients affects the plant and insect community and is especially important for predators like wolf spiders, which in many shore habitats have a diet that is to a large part comprised of insects with aquatic larvae stages, such as chironomids (Mellbrand and Hambäck 2010). Shore communities are also affected by algal detritus (Mellbrand et al. 2011), which is washed up and provides nutrients for a drift-line vegetation, and feces from fish-eating birds defecating on their nesting islands (Kolb et al. 2010). The importance of marine nutrient inflows into the shore ecosystem varies among sites, and is higher in low productive areas, such as sandy or stony beaches. The terrestrial shore ecosystems are in many ways depending on the conditions of the sea and it is not possible to make distinct boundary to the marine shore ecosystem.

## Marine shore ecosystems in the Baltic Sea

The geographical characteristics of the Baltic Sea have large impacts on species composition of the marine environment. The pronounced latitudinal gradient in salinity and temperature limits the spatial distribution of aquatic species. Most marine species have their northern limit in the Northern Quark (Fig. 1), where salinity drops below 4 psu. Important Baltic Sea species such as bladderwrack (*Fucus vesiculosus*), forming large algal belts with many associated species, and blue mussel (*Mytilus edulis*), the most abundant animal species in large parts of the Baltic sea, constituting up to 90 % of the total animal biomass along the Swedish shores of the Baltic Sea proper (Kautsky 1997), have their northernmost outposts close to this area (HELCOM 2012). The projected decrease in salinity in the Bothnian Sea will clearly affect these two species, and may move their northern distribution limit 400 km south (into the Åland Sea). In this case, marine algae will be replaced by freshwater species, the large algal belts will disappear, and consequently the diversity of associated invertebrates will decrease. Similarly, the marine phanerogame *Zostera marina,* forming species-rich sea grass beds, has its salinity limit around 5.5 (northern Stockholm archipelago to southwestern shores of Finland) (Boström et al. 2002). If salinity decreases it will probably be found only at the southern shores of the Baltic Sea, which would have fundamental consequences for many associated species, including many coastal fish species that use sea grass beds as nursing habitats.

Besides changes in salinity, increased water temperatures will probably be the climatic factor that causes the most profound effect on phytobenthic communities in the Baltic Sea. In addition to benefitting warm water species (including non-indigenous and invasive species that are currently temperature limited in the Baltic Sea), higher temperature also increases metabolism (Dillon et al. 2010), which for animals would increase the demand for food and potentially cause food limitation. Blue mussels have decreased dramatically following unusually long periods of high summer temperature, when primary production was also low (Kautsky unpubl. results). The sensitivity of the mussel community to increasing temperature may cause unpredictable ecological changes, as this is a key species for recirculation of nutrients to the Baltic ecosystem (Kautsky and Evans 1987).

The last decades have witnessed major improvements of the phytobenthic communities in large parts of the Baltic Sea as a result of decreased eutrophication (Kautsky 2012). *Fucus* *vesiculosus* belts in the Åland Sea have today the same depth penetration as in the 1940s, after a minimum in the mid-1980s (Jansson and Kautsky 1977; Kautsky 1995). Climate change may reverse this improvement, as increased sea levels may increase shoreline erosion and leakage of soil nutrients and cause increased eutrophication and more narrow algal belts. Further, increasing water temperatures and more available nutrients will increase primary production and thus the turbidity in coastal waters. These factors together tend to increase the occurrence of opportunistic species, such as fast growing filamentous algae (Bergström et al. 2003), which might hamper establishment of perennial species such as *Fucus vesiculosus* (Berger et al. 2003). A change in the algal community will change the content of drift lines washed up onto the shores and indirectly affect the shore plant and insect assemblages. Deposited filamentous algae might not provide the same structural habitat for terrestrial arthropods or the same nutrient source for terrestrial plants and might thus change the species composition in terrestrial shore ecosystems. Baltic shore vegetation is also influenced by eutrophication directly (von Numers and Korvenpää 2007), and changes in shore vegetation would cascade to both herbivore and predator communities.

## Discussion

An improved understanding of climate change impacts in coastal ecosystems necessitates a cross-disciplinary approach that extends to management actions. A problem in the Baltic Sea area is that coastal processes, species, and especially the interaction between aquatic and terrestrial habitats are weakly represented in current monitoring programs and scientific studies. The limited information on hydrological conditions and changes in coastal zones (Hannerz and Destouni 2006) decreases our ability to accurately project climate change effects and spatiotemporal extrapolation. It is fundamental to increase this information in order to understand ongoing and future changes and for society to adapt to them.

Similar to other systems, we would expect changes in the spatial distribution of coastal plants and animals and possibly large consequences of invasive species in Baltic shore ecosystems. We have in this review mainly focused on aspects that are unique to shore ecosystems like the projected changes in sea levels and disturbance regimes. In addition, our focus has been on the Swedish coastline in the central Baltic Sea where the isostatic land uplift has been very important in shaping past and present coastal habitat types. A change from land uplift coast to SLR would cause radical changes in land and sea ecosystem structure. Terrestrial plant species adapted to colonize the virgin land will have less success in establishing in dense vegetation. Most likely, early successional plants may suffer from increased competition, provided that climate change does not also increase disturbance. For instance, it has been suggested that ice drift and ridge formation might increase, and together with stronger winds change the timing and occurrence of ice scouring (HELCOM 2013), which might be beneficial for disturbance-tolerant species. Many species-rich shore meadows will disappear with severe consequences for, not only plants and insects, but also migrating birds.

Shore meadows are important areas not only from a biological perspective, but are also subject to intense land use. While climate change in combination with human impact may cause problems for the biological system, climate change will also affect how people use the coastal systems. Heavy storms and flooding can be major societal disturbances also in developed countries with strong economies and good planning. Projections for the Baltic Sea include flooding of coastal areas, erosion of sandy beaches, and destruction of harbors (Kont et al. 2003). Since coastlines are extremely attractive for human settlement and activities, there are often conflicts between human interest and habitat conservation, which may intensify with climate change. Even though climate change issues are often acknowledged in management responses, the necessary knowledge from different disciplines is often not synchronized and accounted for. It is expected that within the complex governance system in Sweden, coordination is a major challenging issue (de la Torre-Castro 2012). Society needs to include an ecosystem approach and see the complexity of the problem when deciding on measures. To see the complexity, the separation of terrestrial and marine ecosystems and habitats, in both science and decision-making, should be avoided in coastal spatial planning in the future. Considering the scenarios presented in this paper, we distinguish some key issues to improve awareness of and adaptation to climate change in coastal regions of the Baltic Sea.

Firstly, we believe that management responses, institutions and organizations have to match the biophysical realities of the Baltic Sea system. There should be congruence between management administration and the reality. Key issues to be considered are the salinity gradient, changes in land use, hydrological patterns, and local characteristics of the ecosystems. Improved modeling of water flow dynamics in the coastal zone, not separated into either land or sea, would facilitate coastal water resource management and provide a better foundation for studies of ecology and change at the land–sea boundary.

Secondly, there is a need for increased awareness and adaptations. Actions regarding climate change are relatively new; there is a need to speed up the process and to develop consciousness at all levels of society and to implement this knowledge in the decision making process, such as in permits for construction of near-shore buildings. To be better prepared and increase the understanding of how coastal areas might be affected in the future, there is a great need for better and more regional climate models that are shared among government organizations, NGOs and decision makers.

Thirdly, better communication and information exchange is badly needed to speed up the process of creating relevant management actions that matches the biophysical conditions. Multiple actors, such as authorities, scientists, NGO’s, and other representatives of the society can contribute in the information exchange and thus decrease coordination problems and increase the creativity in problem solving.

Finally, climate change adaptation will require resources, not least economic, and the tension between central authority responsibility versus regional and municipality authorities has to be solved.

## Conclusion

In this paper, we have focused on climate change impact on the Swedish coastline of the central Baltic Sea, where increased sea levels would radically change the conditions in terrestrial and marine ecosystems adapted to a land uplift coast. These issues are relevant for other parts of the Baltic Sea, and we believe that the cross-disciplinary approach of the paper is necessary to understand present and future processes in the coastal zone. We stress the importance of an ecosystem approach in scientific studies, monitoring programs, and management of coastal areas without the common separation of terrestrial and marine ecosystems. We further recommend that societal responses to climate change in Baltic coastal ecosystems should match the biophysical realities of the Baltic Sea area. We also believe that better communication and information exchange is badly needed as well as economic support in order to increase the awareness of and adaptation to climate change impact in coastal areas of the Baltic Sea.
